# Trimethyl-3-meth­oxy-4-oxo-5-triphenyl­phospho­ranyl­idene­cyclo­pent-1-ene-1,2,3-tricarboxyl­ate

**DOI:** 10.1107/S1600536810037815

**Published:** 2010-10-09

**Authors:** Krzysztof K. Krawczyk, Krystyna Wojtasiewicz, Jan K. Maurin, Ewa Gronowska, Zbigniew Czarnocki

**Affiliations:** a Faculty of Chemistry, University of Warsaw, Pasteura 1, 02-093 Warsaw, Poland; b National Medicines Institute, Chełmska 30/34, 00-725 Warsaw, Poland; c Institute of Atomic Energy, 05-400 Otwock-Świerk, Poland

## Abstract

The title compound, C_30_H_27_O_8_P (2), was formed as one of two products {(1) [Krawczyk *et al.* (2010 ▶). *Acta Cryst.* E**66** (cv2752)] and (2)} in the reaction of dimethyl acetyl­enedicarboxyl­ate with triphenyl­phosphine. The mol­ecule of (2) consists of a five-membered carbocyclic ring. The P atom is a part of a triphenylphosphoranylidene substituent. In contrast to (1), the five-membered ring of (2) is planar, the r.m.s. deviation being only 0.009 (2) Å.

## Related literature

For a detailed study of adduct formation from triaryl­phosphines and acetyl­enedicarboxyl­ate, see: Waite *et al.* (1971 ▶). For related structures, see: Spek (1987 ▶); Thomas & Hamor (1993 ▶); Krawczyk *et al.* (2010 ▶).
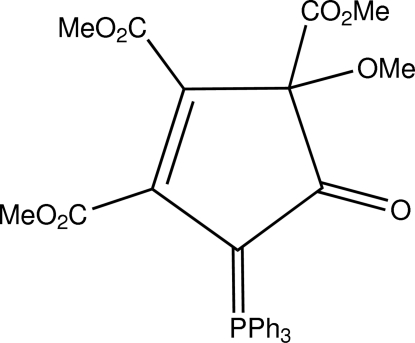


## Experimental

### 

#### Crystal data

C_30_H_27_O_8_P
*M* *_r_* = 546.49
Monoclinic, 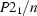

*a* = 10.9220 (1) Å
*b* = 15.1215 (1) Å
*c* = 16.7423 (1) Å
β = 92.145 (1)°
*V* = 2763.17 (4) Å^3^
*Z* = 4
Cu *K*α radiation
μ = 1.31 mm^−1^
*T* = 293 K
0.37 × 0.18 × 0.07 mm

#### Data collection

Oxford Diffraction Xcalibur diffractometer with Ruby CCD
Absorption correction: analytical (*CrysAlis RED*; Oxford Diffraction, 2006 ▶) *T*_min_ = 0.717, *T*_max_ = 0.926
24587 measured reflections
5014 independent reflections
3667 reflections with *I* > 2σ(*I*)
*R*_int_ = 0.043

#### Refinement

*R*[*F* ^2^ > 2σ(*F* ^2^)] = 0.047
*wR*(*F* ^2^) = 0.129
*S* = 1.01
5014 reflections
352 parameters
H-atom parameters constrained
Δρ_max_ = 0.41 e Å^−3^
Δρ_min_ = −0.21 e Å^−3^

### 

Data collection: *CrysAlis CCD* (Oxford Diffraction, 2006 ▶); cell refinement: *CrysAlis RED* (Oxford Diffraction, 2006 ▶); data reduction: *CrysAlis RED*; program(s) used to solve structure: *SHELXS97* (Sheldrick, 2008 ▶); program(s) used to refine structure: *SHELXL97* (Sheldrick, 2008 ▶); molecular graphics: *SHELXTL-NT* (Sheldrick, 2008 ▶); software used to prepare material for publication: *SHELXL97*.

## Supplementary Material

Crystal structure: contains datablocks paper, I. DOI: 10.1107/S1600536810037815/cv2753sup1.cif

Structure factors: contains datablocks I. DOI: 10.1107/S1600536810037815/cv2753Isup2.hkl

Additional supplementary materials:  crystallographic information; 3D view; checkCIF report

## Figures and Tables

**Fig. 1.**
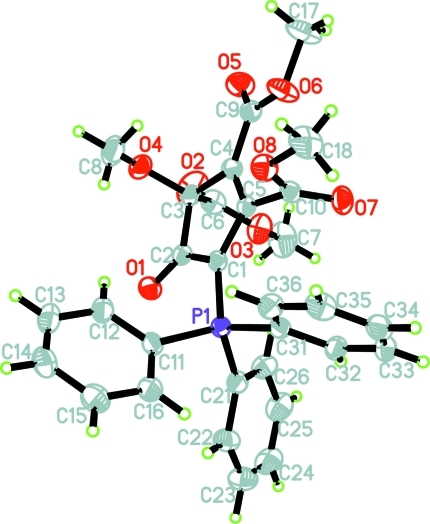
Molecular structure of (2) showing the atomi labelling and 30% probability displacement ellipsoids.

## supplementary crystallographic information

### Comment

Trimethyl-3-methoxy-4-oxo-5-triphenylphosphoranylidenecyclopent-1-ene-1,2,3- tricarboxylate (2) is one of two 1:2 adducts formed as the minor compound in the reaction of triphenylphosphine and acetylenedicarboxylate, described already in 1971 (Waite *et al.*). By using dry toluene instead of diethyl ether, and by reducing the temperature of the reaction to -78%C we menaged to raise the yield of the reaction from 21% to 28%. Crystal structure of the other compound - tetramethyl 1,1,2-triphenyl-2*H*-1λ^5^-phosphole-2,3,4,5-tetracarboxylate was also published recently (Krawczyk *et al.*, 2010). In the present comunication we report on the crystal structure of compound (2).

In molecule (2) (Fig. 1), one of the acetyl groups at C4 is almost co-planar with the five-membered ring with a dihedral angle of 8.60 (3)° whereas all other acetyl and methoxy groups at C3 and C5 atoms are perpendicular to it with the dihedral angles of 86.31 (14), 84.95 (12) and 89.09 (9)°, respectively. The phenyl rings bonded to the phosphorous atom in (2) have similar conformations to that observed at room temperature for the parent triphenylphosphine in both polymorphic structures (Spek, 1987; Thomas & Hamor, 1993) assuring the lowest repulsion of the neighboring fragments.

### Experimental

A mixture of acetylenedicarboxylate (0.5 g, 3,52 mmol) in 3 ml of dry toluene was placed in a two-neck round bottom flask, and cooled to -78°C (solid CO_2_/acetone bath) with stirring. The solution of triphenylphosphine (0.47 g, 1.80 mmol) in 3 ml of dry toluene was then added dropwise under argon during 20 min. The reaction was then left to reach slowly room temperature overnight. After evaporation of the solvent under reduced pressure, the remaining oil was dissolved in ethyl acetate and purified by column chromatography (Merck silica gel, 230 - 400 mesh, ethyl acetate, and then ethyl acetate/methanol 19:1 as eluent). Both products could be easily recrystallized from ethyl acetate/diethyl ether. The 2*H*-phosphole 1 (0.61 g, 63%) had Rf = 0.46 (ethyl acetate) and a melting point of 253–255°C (Waite, *et al.*1971). The second eluted product - (2) (0.27 g, 28%) - showed a green fluorescence in UV light (λ = 365 nm), had Rf = 0.18 (ethyl acetate) and melted at 243–244°C [(Waite *et al.*, 1971), m.p. 222–224°C]. The single-crystal of (2) was obtained by slow evaporation of its ethyl acetate/diethyl ether solution.

### Refinement

H atoms were placed in calcluated positions and were included in the refinement with *U*_iso_(H) = 1.2*U*_eq_(C) [1.5 in the case of methyl groups H atoms]. Isotropic displacement parameters for hydrogen atoms bonded to either oxygen or nitrogen atoms were refined independently.

### Crystal data

**Table:**

C_30_H_27_O_8_P	*F*(000) = 1144
*M**_r_* = 546.49	*D*_x_ = 1.314 Mg m^−^^3^
Monoclinic, *P*2_1_/*n*	Cu *K*α radiation, λ = 1.54178 Å
*a* = 10.9220 (1) Å	Cell parameters from 8837 reflections
*b* = 15.1215 (1) Å	θ = 2.6–70.3°
*c* = 16.7423 (1) Å	µ = 1.31 mm^−^^1^
β = 92.145 (1)°	*T* = 293 K
*V* = 2763.17 (4) Å^3^	Parallelepiped, colourless
*Z* = 4	0.37 × 0.18 × 0.07 mm

### Data collection

**Table:**

Oxford Diffraction Xcalibur diffractometer with Ruby CCD	5014 independent reflections
Radiation source: fine-focus sealed tube	3667 reflections with *I* > 2σ(*I*)
graphite	*R*_int_ = 0.043
ο and φ scans	θ_max_ = 70.4°, θ_min_ = 3.9°
Absorption correction: analytical (*CrysAlis RED*; Oxford Diffraction, 2006)	*h* = −12→13
*T*_min_ = 0.717, *T*_max_ = 0.926	*k* = −17→18
24587 measured reflections	*l* = −20→19

### Refinement

**Table:**

Refinement on *F*^2^	Primary atom site location: structure-invariant direct methods
Least-squares matrix: full	Secondary atom site location: difference Fourier map
*R*[*F*^2^ > 2σ(*F*^2^)] = 0.047	Hydrogen site location: inferred from neighbouring sites
*wR*(*F*^2^) = 0.129	H-atom parameters constrained
*S* = 1.01	*w* = 1/[σ^2^(*F*_o_^2^) + (0.0824*P*)^2^] where *P* = (*F*_o_^2^ + 2*F*_c_^2^)/3
5014 reflections	(Δ/σ)_max_ < 0.001
352 parameters	Δρ_max_ = 0.41 e Å^−^^3^
0 restraints	Δρ_min_ = −0.21 e Å^−^^3^

### Special details

**Table:**

Experimental. Analytical numeric absorption correction using a multifaceted crystal model based on expressions derived by R.C. Clark & J.S. Reid. (Clark, R. C. & Reid, J. S. (1995). Acta Cryst. A51, 887–897)
Geometry. All e.s.d.'s (except the e.s.d. in the dihedral angle between two l.s. planes) are estimated using the full covariance matrix. The cell e.s.d.'s are taken into account individually in the estimation of e.s.d.'s in distances, angles and torsion angles; correlations between e.s.d.'s in cell parameters are only used when they are defined by crystal symmetry. An approximate (isotropic) treatment of cell e.s.d.'s is used for estimating e.s.d.'s involving l.s. planes.
Refinement. Refinement of *F*^2^ against ALL reflections. The weighted *R*-factor *wR* and goodness of fit *S* are based on *F*^2^, conventional *R*-factors *R* are based on *F*, with *F* set to zero for negative *F*^2^. The threshold expression of *F*^2^ > σ(*F*^2^) is used only for calculating *R*-factors(gt) *etc*. and is not relevant to the choice of reflections for refinement. *R*-factors based on *F*^2^ are statistically about twice as large as those based on *F*, and *R*- factors based on ALL data will be even larger.

### Fractional atomic coordinates and isotropic or equivalent isotropic displacement parameters (Å^2^)

**Table:**

	*x*	*y*	*z*	*U*_iso_*/*U*_eq_	
P1	0.98130 (5)	0.33555 (3)	0.23236 (3)	0.03465 (16)	
O1	0.78889 (16)	0.31389 (11)	0.08918 (8)	0.0499 (4)	
O2	0.4783 (2)	0.40415 (17)	0.08401 (15)	0.0969 (8)	
O3	0.6135 (2)	0.47924 (12)	0.15999 (11)	0.0676 (5)	
O4	0.55002 (18)	0.25150 (12)	0.14528 (10)	0.0587 (5)	
O5	0.40520 (18)	0.34126 (14)	0.28230 (12)	0.0676 (5)	
O6	0.52975 (17)	0.33087 (15)	0.39062 (10)	0.0690 (6)	
O7	0.78341 (18)	0.41525 (11)	0.41526 (9)	0.0579 (5)	
O8	0.78057 (17)	0.26709 (11)	0.41634 (8)	0.0519 (4)	
C1	0.8225 (2)	0.33213 (13)	0.23123 (11)	0.0344 (4)	
C2	0.7528 (2)	0.32327 (12)	0.15769 (12)	0.0373 (5)	
C3	0.6140 (2)	0.32578 (14)	0.17661 (12)	0.0396 (5)	
C4	0.6183 (2)	0.33365 (13)	0.26716 (12)	0.0392 (5)	
C5	0.7364 (2)	0.33655 (12)	0.29464 (11)	0.0342 (4)	
C6	0.5565 (2)	0.40552 (18)	0.13463 (14)	0.0541 (6)	
C7	0.5738 (4)	0.5607 (2)	0.1224 (2)	0.0985 (13)	
H7A	0.6202	0.6091	0.1450	0.148*	
H7B	0.4883	0.5698	0.1311	0.148*	
H7C	0.5865	0.5575	0.0660	0.148*	
C8	0.5827 (4)	0.17164 (18)	0.18298 (19)	0.0814 (10)	
H8A	0.5359	0.1242	0.1590	0.122*	
H8B	0.5661	0.1754	0.2388	0.122*	
H8C	0.6684	0.1607	0.1769	0.122*	
C9	0.5071 (2)	0.33579 (15)	0.31160 (13)	0.0440 (5)	
C10	0.7700 (2)	0.34554 (14)	0.38231 (11)	0.0390 (5)	
C11	1.0463 (2)	0.22934 (14)	0.20702 (12)	0.0419 (5)	
C12	0.9720 (3)	0.16379 (15)	0.17490 (15)	0.0522 (6)	
H12	0.8905	0.1758	0.1611	0.063*	
C13	1.0193 (3)	0.07979 (18)	0.16328 (18)	0.0683 (8)	
H13	0.9692	0.0355	0.1416	0.082*	
C14	1.1395 (3)	0.06166 (18)	0.18364 (19)	0.0720 (8)	
H14	1.1704	0.0049	0.1770	0.086*	
C15	1.2136 (3)	0.1272 (2)	0.2137 (2)	0.0718 (8)	
H15	1.2955	0.1150	0.2264	0.086*	
C16	1.1685 (2)	0.21181 (17)	0.22555 (16)	0.0558 (6)	
H16	1.2197	0.2562	0.2457	0.067*	
C17	0.4260 (3)	0.3308 (3)	0.4409 (2)	0.0909 (11)	
H17A	0.4539	0.3273	0.4959	0.136*	
H17B	0.3749	0.2807	0.4280	0.136*	
H17C	0.3800	0.3842	0.4325	0.136*	
C18	0.7974 (3)	0.2668 (2)	0.50317 (14)	0.0760 (9)	
H18A	0.8041	0.2070	0.5219	0.114*	
H18B	0.7284	0.2948	0.5265	0.114*	
H18C	0.8708	0.2986	0.5182	0.114*	
C21	1.0310 (2)	0.42131 (14)	0.16544 (11)	0.0427 (5)	
C22	1.1493 (3)	0.42166 (18)	0.13731 (15)	0.0590 (7)	
H22	1.2028	0.3754	0.1499	0.071*	
C23	1.1869 (3)	0.4911 (2)	0.09070 (18)	0.0762 (9)	
H23	1.2657	0.4911	0.0714	0.091*	
C24	1.1094 (3)	0.5600 (2)	0.07247 (17)	0.0731 (9)	
H24	1.1357	0.6063	0.0408	0.088*	
C25	0.9937 (3)	0.56107 (18)	0.10071 (17)	0.0685 (8)	
H25	0.9417	0.6083	0.0887	0.082*	
C26	0.9535 (3)	0.49155 (16)	0.14749 (14)	0.0554 (6)	
H26	0.8746	0.4923	0.1667	0.066*	
C31	1.04400 (19)	0.36215 (14)	0.33050 (11)	0.0362 (5)	
C32	1.0687 (2)	0.44928 (14)	0.35069 (13)	0.0446 (5)	
H32	1.0548	0.4936	0.3129	0.053*	
C33	1.1136 (2)	0.47116 (18)	0.42599 (14)	0.0542 (6)	
H33	1.1294	0.5299	0.4392	0.065*	
C34	1.1349 (2)	0.40574 (19)	0.48145 (14)	0.0566 (7)	
H34	1.1658	0.4204	0.5323	0.068*	
C35	1.1113 (3)	0.31865 (19)	0.46293 (14)	0.0583 (7)	
H35	1.1263	0.2749	0.5011	0.070*	
C36	1.0653 (2)	0.29617 (16)	0.38761 (13)	0.0469 (5)	
H36	1.0486	0.2374	0.3751	0.056*	

### Atomic displacement parameters (Å^2^)

**Table:**

	*U*^11^	*U*^22^	*U*^33^	*U*^12^	*U*^13^	*U*^23^
P1	0.0370 (3)	0.0358 (3)	0.0312 (3)	−0.0019 (2)	0.0013 (2)	−0.00179 (19)
O1	0.0546 (11)	0.0653 (10)	0.0298 (7)	−0.0056 (8)	0.0028 (7)	−0.0040 (6)
O2	0.0973 (19)	0.1003 (17)	0.0891 (15)	−0.0024 (14)	−0.0496 (15)	0.0148 (13)
O3	0.0841 (15)	0.0491 (10)	0.0682 (11)	0.0071 (9)	−0.0154 (10)	0.0070 (8)
O4	0.0622 (12)	0.0619 (10)	0.0516 (9)	−0.0204 (9)	−0.0027 (8)	−0.0095 (7)
O5	0.0404 (11)	0.0935 (14)	0.0688 (11)	−0.0029 (10)	−0.0008 (9)	−0.0047 (10)
O6	0.0470 (11)	0.1185 (16)	0.0423 (9)	0.0043 (11)	0.0120 (8)	0.0006 (9)
O7	0.0722 (13)	0.0582 (10)	0.0432 (8)	−0.0052 (9)	−0.0007 (8)	−0.0150 (7)
O8	0.0619 (12)	0.0585 (9)	0.0349 (7)	0.0014 (8)	−0.0024 (7)	0.0075 (7)
C1	0.0373 (12)	0.0367 (10)	0.0291 (9)	−0.0007 (9)	−0.0006 (8)	−0.0021 (7)
C2	0.0421 (12)	0.0354 (10)	0.0342 (10)	−0.0051 (9)	−0.0023 (9)	−0.0002 (8)
C3	0.0385 (12)	0.0473 (11)	0.0327 (10)	−0.0073 (10)	−0.0048 (9)	−0.0029 (8)
C4	0.0399 (13)	0.0415 (11)	0.0360 (10)	−0.0016 (10)	0.0006 (9)	−0.0009 (8)
C5	0.0375 (12)	0.0326 (9)	0.0323 (9)	−0.0016 (9)	−0.0009 (9)	−0.0021 (7)
C6	0.0502 (16)	0.0706 (17)	0.0405 (12)	0.0056 (13)	−0.0100 (11)	0.0057 (11)
C7	0.145 (4)	0.0617 (18)	0.088 (2)	0.022 (2)	−0.009 (2)	0.0175 (16)
C8	0.120 (3)	0.0521 (16)	0.0733 (18)	−0.0230 (17)	0.0140 (19)	−0.0109 (13)
C9	0.0382 (13)	0.0468 (12)	0.0469 (12)	−0.0011 (10)	0.0004 (10)	−0.0039 (9)
C10	0.0378 (12)	0.0474 (12)	0.0316 (10)	0.0003 (10)	0.0005 (9)	−0.0027 (9)
C11	0.0453 (14)	0.0412 (11)	0.0398 (10)	0.0030 (10)	0.0077 (10)	−0.0050 (8)
C12	0.0517 (15)	0.0456 (12)	0.0594 (14)	0.0004 (11)	0.0013 (12)	−0.0117 (10)
C13	0.073 (2)	0.0461 (14)	0.0859 (19)	−0.0030 (14)	0.0050 (16)	−0.0171 (13)
C14	0.076 (2)	0.0473 (14)	0.094 (2)	0.0142 (14)	0.0189 (17)	−0.0128 (13)
C15	0.0529 (17)	0.0661 (17)	0.097 (2)	0.0155 (15)	0.0097 (15)	−0.0092 (15)
C16	0.0432 (15)	0.0530 (14)	0.0713 (16)	0.0022 (12)	0.0050 (12)	−0.0120 (12)
C17	0.070 (2)	0.136 (3)	0.0689 (19)	0.002 (2)	0.0368 (17)	0.0042 (19)
C18	0.094 (3)	0.098 (2)	0.0358 (12)	0.0006 (19)	−0.0035 (14)	0.0162 (13)
C21	0.0521 (15)	0.0436 (11)	0.0325 (10)	−0.0067 (10)	0.0022 (10)	0.0006 (8)
C22	0.0573 (17)	0.0634 (15)	0.0572 (14)	−0.0048 (13)	0.0148 (12)	0.0041 (12)
C23	0.077 (2)	0.084 (2)	0.0702 (18)	−0.0189 (18)	0.0276 (16)	0.0059 (15)
C24	0.095 (3)	0.0680 (18)	0.0565 (15)	−0.0271 (17)	0.0095 (16)	0.0160 (13)
C25	0.086 (2)	0.0552 (15)	0.0634 (16)	−0.0090 (15)	−0.0081 (16)	0.0184 (12)
C26	0.0581 (17)	0.0534 (14)	0.0544 (14)	−0.0054 (12)	−0.0013 (12)	0.0105 (11)
C31	0.0326 (11)	0.0422 (10)	0.0337 (9)	−0.0007 (9)	0.0004 (9)	−0.0013 (8)
C32	0.0487 (14)	0.0420 (11)	0.0429 (11)	−0.0054 (10)	0.0002 (10)	−0.0030 (9)
C33	0.0557 (16)	0.0598 (14)	0.0472 (13)	−0.0084 (12)	0.0027 (11)	−0.0156 (11)
C34	0.0478 (15)	0.0851 (18)	0.0366 (11)	−0.0052 (13)	−0.0011 (11)	−0.0119 (11)
C35	0.0570 (17)	0.0771 (18)	0.0405 (12)	0.0070 (14)	−0.0010 (11)	0.0122 (11)
C36	0.0513 (15)	0.0487 (12)	0.0405 (11)	−0.0028 (11)	−0.0022 (10)	0.0032 (9)

### Geometric parameters (Å, °)

**Table:**

P1—C1	1.734 (2)	C13—H13	0.9300
P1—C31	1.802 (2)	C14—C15	1.363 (4)
P1—C21	1.810 (2)	C14—H14	0.9300
P1—C11	1.813 (2)	C15—C16	1.389 (4)
O1—C2	1.235 (2)	C15—H15	0.9300
O2—C6	1.181 (3)	C16—H16	0.9300
O3—C6	1.338 (3)	C17—H17A	0.9600
O3—C7	1.443 (3)	C17—H17B	0.9600
O4—C8	1.403 (4)	C17—H17C	0.9600
O4—C3	1.413 (3)	C18—H18A	0.9600
O5—C9	1.202 (3)	C18—H18B	0.9600
O6—C9	1.339 (3)	C18—H18C	0.9600
O6—C17	1.437 (3)	C21—C26	1.384 (4)
O7—C10	1.196 (3)	C21—C22	1.393 (4)
O8—C10	1.319 (3)	C22—C23	1.380 (4)
O8—C18	1.458 (3)	C22—H22	0.9300
C1—C2	1.430 (3)	C23—C24	1.370 (5)
C1—C5	1.446 (3)	C23—H23	0.9300
C2—C3	1.561 (3)	C24—C25	1.366 (5)
C3—C4	1.520 (3)	C24—H24	0.9300
C3—C6	1.519 (3)	C25—C26	1.392 (4)
C4—C5	1.355 (3)	C25—H25	0.9300
C4—C9	1.448 (3)	C26—H26	0.9300
C5—C10	1.506 (3)	C31—C32	1.384 (3)
C7—H7A	0.9600	C31—C36	1.395 (3)
C7—H7B	0.9600	C32—C33	1.376 (3)
C7—H7C	0.9600	C32—H32	0.9300
C8—H8A	0.9600	C33—C34	1.371 (4)
C8—H8B	0.9600	C33—H33	0.9300
C8—H8C	0.9600	C34—C35	1.375 (4)
C11—C12	1.378 (3)	C34—H34	0.9300
C11—C16	1.384 (3)	C35—C36	1.382 (3)
C12—C13	1.388 (4)	C35—H35	0.9300
C12—H12	0.9300	C36—H36	0.9300
C13—C14	1.372 (4)		
			
C1—P1—C31	111.24 (9)	C15—C14—C13	119.7 (3)
C1—P1—C21	109.71 (10)	C15—C14—H14	120.1
C31—P1—C21	106.98 (10)	C13—C14—H14	120.1
C1—P1—C11	111.81 (10)	C14—C15—C16	120.9 (3)
C31—P1—C11	105.80 (10)	C14—C15—H15	119.6
C21—P1—C11	111.15 (10)	C16—C15—H15	119.6
C6—O3—C7	116.4 (2)	C11—C16—C15	119.3 (2)
C8—O4—C3	113.8 (2)	C11—C16—H16	120.4
C9—O6—C17	117.3 (2)	C15—C16—H16	120.4
C10—O8—C18	116.02 (19)	O6—C17—H17A	109.5
C2—C1—C5	107.24 (18)	O6—C17—H17B	109.5
C2—C1—P1	120.83 (15)	H17A—C17—H17B	109.5
C5—C1—P1	131.93 (15)	O6—C17—H17C	109.5
O1—C2—C1	129.2 (2)	H17A—C17—H17C	109.5
O1—C2—C3	122.51 (19)	H17B—C17—H17C	109.5
C1—C2—C3	108.26 (16)	O8—C18—H18A	109.5
O4—C3—C4	115.39 (17)	O8—C18—H18B	109.5
O4—C3—C6	105.61 (18)	H18A—C18—H18B	109.5
C4—C3—C6	113.33 (18)	O8—C18—H18C	109.5
O4—C3—C2	112.08 (17)	H18A—C18—H18C	109.5
C4—C3—C2	102.15 (17)	H18B—C18—H18C	109.5
C6—C3—C2	108.24 (18)	C26—C21—C22	119.4 (2)
C5—C4—C9	129.16 (19)	C26—C21—P1	119.29 (18)
C5—C4—C3	109.55 (18)	C22—C21—P1	121.04 (19)
C9—C4—C3	121.3 (2)	C23—C22—C21	119.6 (3)
C4—C5—C1	112.75 (17)	C23—C22—H22	120.2
C4—C5—C10	121.88 (18)	C21—C22—H22	120.2
C1—C5—C10	125.35 (19)	C24—C23—C22	120.7 (3)
O2—C6—O3	123.9 (3)	C24—C23—H23	119.6
O2—C6—C3	126.4 (3)	C22—C23—H23	119.6
O3—C6—C3	109.6 (2)	C25—C24—C23	120.2 (3)
O3—C7—H7A	109.5	C25—C24—H24	119.9
O3—C7—H7B	109.5	C23—C24—H24	119.9
H7A—C7—H7B	109.5	C24—C25—C26	120.1 (3)
O3—C7—H7C	109.5	C24—C25—H25	120.0
H7A—C7—H7C	109.5	C26—C25—H25	120.0
H7B—C7—H7C	109.5	C21—C26—C25	119.9 (3)
O4—C8—H8A	109.5	C21—C26—H26	120.1
O4—C8—H8B	109.5	C25—C26—H26	120.1
H8A—C8—H8B	109.5	C32—C31—C36	119.16 (19)
O4—C8—H8C	109.5	C32—C31—P1	119.95 (16)
H8A—C8—H8C	109.5	C36—C31—P1	120.87 (16)
H8B—C8—H8C	109.5	C33—C32—C31	120.8 (2)
O5—C9—O6	122.8 (2)	C33—C32—H32	119.6
O5—C9—C4	125.0 (2)	C31—C32—H32	119.6
O6—C9—C4	112.2 (2)	C34—C33—C32	119.5 (2)
O7—C10—O8	125.88 (19)	C34—C33—H33	120.2
O7—C10—C5	123.38 (19)	C32—C33—H33	120.2
O8—C10—C5	110.73 (17)	C33—C34—C35	120.8 (2)
C12—C11—C16	119.9 (2)	C33—C34—H34	119.6
C12—C11—P1	119.90 (19)	C35—C34—H34	119.6
C16—C11—P1	120.01 (17)	C34—C35—C36	120.0 (2)
C11—C12—C13	119.8 (3)	C34—C35—H35	120.0
C11—C12—H12	120.1	C36—C35—H35	120.0
C13—C12—H12	120.1	C35—C36—C31	119.7 (2)
C14—C13—C12	120.4 (3)	C35—C36—H36	120.2
C14—C13—H13	119.8	C31—C36—H36	120.2
C12—C13—H13	119.8		
			
C31—P1—C1—C2	172.06 (15)	C18—O8—C10—C5	−172.4 (2)
C21—P1—C1—C2	53.89 (18)	C4—C5—C10—O7	−89.4 (3)
C11—P1—C1—C2	−69.89 (18)	C1—C5—C10—O7	89.1 (3)
C31—P1—C1—C5	−8.1 (2)	C4—C5—C10—O8	89.8 (2)
C21—P1—C1—C5	−126.25 (19)	C1—C5—C10—O8	−91.7 (2)
C11—P1—C1—C5	110.0 (2)	C1—P1—C11—C12	12.7 (2)
C5—C1—C2—O1	−177.0 (2)	C31—P1—C11—C12	133.97 (19)
P1—C1—C2—O1	2.9 (3)	C21—P1—C11—C12	−110.25 (19)
C5—C1—C2—C3	2.1 (2)	C1—P1—C11—C16	−162.32 (18)
P1—C1—C2—C3	−177.97 (14)	C31—P1—C11—C16	−41.1 (2)
C8—O4—C3—C4	−48.1 (3)	C21—P1—C11—C16	74.7 (2)
C8—O4—C3—C6	−174.1 (2)	C16—C11—C12—C13	1.7 (4)
C8—O4—C3—C2	68.3 (3)	P1—C11—C12—C13	−173.3 (2)
O1—C2—C3—O4	53.3 (3)	C11—C12—C13—C14	0.1 (4)
C1—C2—C3—O4	−125.94 (18)	C12—C13—C14—C15	−1.6 (5)
O1—C2—C3—C4	177.36 (18)	C13—C14—C15—C16	1.3 (5)
C1—C2—C3—C4	−1.8 (2)	C12—C11—C16—C15	−2.0 (4)
O1—C2—C3—C6	−62.8 (2)	P1—C11—C16—C15	173.1 (2)
C1—C2—C3—C6	118.00 (19)	C14—C15—C16—C11	0.4 (4)
O4—C3—C4—C5	122.7 (2)	C1—P1—C21—C26	24.4 (2)
C6—C3—C4—C5	−115.4 (2)	C31—P1—C21—C26	−96.3 (2)
C2—C3—C4—C5	0.8 (2)	C11—P1—C21—C26	148.61 (18)
O4—C3—C4—C9	−56.5 (3)	C1—P1—C21—C22	−161.12 (19)
C6—C3—C4—C9	65.5 (3)	C31—P1—C21—C22	78.1 (2)
C2—C3—C4—C9	−178.30 (18)	C11—P1—C21—C22	−36.9 (2)
C9—C4—C5—C1	179.5 (2)	C26—C21—C22—C23	−1.3 (4)
C3—C4—C5—C1	0.5 (2)	P1—C21—C22—C23	−175.7 (2)
C9—C4—C5—C10	−1.8 (3)	C21—C22—C23—C24	0.6 (5)
C3—C4—C5—C10	179.16 (17)	C22—C23—C24—C25	0.3 (5)
C2—C1—C5—C4	−1.7 (2)	C23—C24—C25—C26	−0.7 (5)
P1—C1—C5—C4	178.44 (16)	C22—C21—C26—C25	0.9 (4)
C2—C1—C5—C10	179.66 (18)	P1—C21—C26—C25	175.5 (2)
P1—C1—C5—C10	−0.2 (3)	C24—C25—C26—C21	0.0 (4)
C7—O3—C6—O2	0.6 (4)	C1—P1—C31—C32	−92.5 (2)
C7—O3—C6—C3	176.8 (3)	C21—P1—C31—C32	27.3 (2)
O4—C3—C6—O2	−2.9 (4)	C11—P1—C31—C32	145.84 (19)
C4—C3—C6—O2	−130.1 (3)	C1—P1—C31—C36	85.8 (2)
C2—C3—C6—O2	117.3 (3)	C21—P1—C31—C36	−154.43 (19)
O4—C3—C6—O3	−178.97 (19)	C11—P1—C31—C36	−35.8 (2)
C4—C3—C6—O3	53.8 (3)	C36—C31—C32—C33	0.0 (4)
C2—C3—C6—O3	−58.8 (2)	P1—C31—C32—C33	178.32 (18)
C17—O6—C9—O5	1.1 (4)	C31—C32—C33—C34	0.5 (4)
C17—O6—C9—C4	−178.9 (3)	C32—C33—C34—C35	−0.4 (4)
C5—C4—C9—O5	172.6 (2)	C33—C34—C35—C36	−0.1 (4)
C3—C4—C9—O5	−8.4 (3)	C34—C35—C36—C31	0.5 (4)
C5—C4—C9—O6	−7.4 (3)	C32—C31—C36—C35	−0.5 (4)
C3—C4—C9—O6	171.55 (19)	P1—C31—C36—C35	−178.79 (19)
C18—O8—C10—O7	6.8 (4)		
